# 

**Published:** 1896-10

**Authors:** 


### BOOKS RECEIVED.

A System of Surgery. In contributions by twenty-five English authors. Edited by Frederick Treves, F. R. C. S., Surgeon to, and Lecturer on, Surgery at the London Hospital ; Examiner in Surgery at the University of Cambridge. Volume II. In one octavo volume of 1120 pages, with two colored plates and 487 illustrations. Cloth, $8.00. Philadelphia and New York : Lea Brothers & Co.

An American Text-Book of Applied Therapeutics for the Use of Practitioners and Students. Edited by J. C. Wilson, M. D., Professor of the Practice of Medicine and Clinical Medicine in the Jefferson Medical College ; Attending Physician to the Hospital of the Jefferson Medical College, to the German Hospital, and to the Pennsylvania Hospital, Philadelphia. Assisted by Augustus A. Eshner, M. D. Professor of Clinical Medicine in the Philadelphia Polyclinic ; Attending Physician to the Philadelphia Hospital. Octavo, pp. 1326. Illustrated. Price, cloth, $7.00 ; sheepskin and half Morocco, $8.00 ; half Russia, $9.50. Philadelphia: W. B. Saunders, 925 Walnut street. 1896.

A Handbook of Pathological Anatomy and Histology. With an introductory section on post mortem examinations and the methods of preserving and examining diseased tissues. By Francis Delafield,

M. D., LL. D., Professor of the Practice of Medicine, College of Physicians and Surgeons, Columbia College, New York, and T. Mitchell Prudden, M. D., Professor of Pathology and Director of the Laboratories of Histology, Pathology and Bacteriology, College of Physicians and Surgeons, Columbia College, New York. Fifth edition. Octavo, pp. viii.—846. Illustrated by 365 wood-engravings, printed in black and colors. New York: William Wood & Co. 1896.

The Medical and Surgical Uses of Electricity. By A. D. Rockwell, A. M., M. D., formerly Professor of Electro-Therapeutics in the New York Post-Graduate Medical School and Hospital, etc. New edition. Octavo, pp. xvi.—612. Illustrated with 200 engravings. Price, $4.50. New York: William Wood & Co. 1896.

Public Health Papers and Reports. Volume XX. Presented at the twenty-second annual meeting of the American Public Health Association, Montreal, Canada, September 25–28, 1894. Together with other papers and an abstract of the records and proceedings. Quarterly series, Volume I. Irving A. Watson, M. D., Secretary. Concord: Republican Press Association. 1895.

A Manual of Pharmacology and Therapeutics. By William Murrell, M. D., F. R. C. P., Physician to, and Lecturer on, Pharmacology and Therapeutics at the Westminster Hospital, etc., etc. Revised by Frederick A. Castle, M. D., Member of the committee for revision and publication of the Pharmacopeia of the United States of America, etc., etc. Octavo, pp. 516. New York: William Wood & Co. 1896.

Functional Disorders of the Nervous System in Women. By T. J. McGillicuddy, A. M., M. D., Consulting Physician to the Italian Hospital, New York, etc., etc. Small octavo, pp. 367. Illustrated. New York: William Wood & Co. 1896.

Eleventh Annual Report of the State Board of Health and Vital Statistics of the Commonwealth of Pennsylvania. Transmitted to the Governor, December 1, 1895. Clarence M. Busch, State Printer of Pennsylvania. 1896.

Index-Catalogue of the Library of the Surgeon-General's Office, United States Army. Authors and Subjects. Second series, Volume I. A—Azzurri. Washington: Government Printing Office. 1896.

A Vest-Pocket Medical Dictionary. Embracing those terms and abbreviations which are commonly found in the medical literature of the day, but excluding the names of drugs and of many special anatomical terms By Albert H. Buck, M. D., New York City. New York: William Wood & Co. 1896.

Feeding in Early Infancy. By Arthur V. Meigs, M. D. Price, 25 cents. Philadelphia: W. B. Saunders. 1896.

The Student's Medical Dictionary. Including all the words and phrases generally used in medicine, with their proper pronunciation and definitions. Based on recent medical literature. By George M. Gould, A. M., M. D., Author of An illustrated dictionary of medicine, biology and allied sciences, etc., etc. With elaborate tables of the bacilli, micrococci, leucomains, ptomains, etc.; of the arteries, ganglia, muscles and nerves; of weights and measures, etc., etc. Tenth edition, rewritten and enlarged. Price, $3.25. Philadelphia: P. Blakiston, Son & Co., 1012 Walnut street. 1896.

Minor Surgery and Bandaging. By Henry R. Wharton, M. D., Demonstrator of Surgery in the University of Pennsylvania. New (3d) edition. In one 12mo volume of 594 pages, with 475 engravings, many being photographic. Cloth, $3.00. Philadelphia: Lea Brothers & Co. 1896.

A Manual of Clinical Diagnosis by Microscopical and Chemical Methods. For students, hospital physicians and practitioners. By Charles E. Simon, M. D., Late Assistant Resident Physician Johns Hopkins Hospital, Baltimore. In one octavo volume of 504 pages, with 132 engravings and ten full-page colored plates. Cloth, $3.50. Philadelphia and New York: Lea Brothers & Co. 1896.

Food in Health and Disease. By I. Burney Yeo, M. D., F. R. C. P., Professor of Therapeutics in King's College, London. New (2d) edition. In one 12mo volume of 592 pages, with four engravings. Cloth, $2.50. Philadelphia and New York: Lea Brothers & Co., Publishers. 1896.

The Ready Reference Handbook of Diseases of the Skin. By George Thomas Jackson, M. D., Professor of Dermatology, Woman's Medical College of the New York Infirmary and in the University of Vermont; Chief of Clinic and Instructor in Dermatology, College of Physicians and Surgeons, New York. New (2d) edition. In one 12mo volume of 589 pages, with sixty-nine illustrations and a colored plate. Cloth, $2.75. Philadelphia: Lea Brothers & Co. 1896.

A Manual of Materia Medica and Pharmacology. Comprising all organic and inorganic drugs, which are and have been official in the United States Pharmacopeia, together with important allied species and useful synthetics. For students of medicine, druggists, pharmacists and physicians. By David M. R. Culbreth, M. D., Professor of Botany, Materia Medica and Pharmacognosy in the Maryland College of Pharmacy, Baltimore. In one octavo volume of 812 pages, with 445 illustrations. Cloth, $4.75. Philadelphia and New York: Lea Brothers & Co., Publishers. 1896.

Anatomy, Descriptive and Surgical. By Henry Gray, F. R. S., Lecturer on Anatomy at St. George's Hospital, London. New and thoroughly revised American edition, much enlarged in text, and in engravings, both colored and black. In one imperial octavo volume of 1239 pages, with 772 large and elaborate engravings on wood. Price of edition with illustrations in colors, cloth, $7.00; leather, $8.00. Price of edition with illustrations in black, cloth, $6.00; leather, $7.00. Philadelphia and New York: Lea Brothers & Co., Publishers. 1896.

A Manual of Venereal Diseases. By James R. Hayden, M. D., Chief of Venereal Clinic, College of Physicians and Surgeons, New York; Professor of Genito-Urinary and Venereal Diseases in the Medical Department of the University of Vermont, etc. In one 12mo volume of 263 pages, with forty-seven engravings. Cloth, $1.50. Philadelphia and New York: Lea Brothers & Co., Publishers. 1896.

Ptomains, Leucomains, Toxins and Antitoxins; or the Chemical Factors in the Causation of Disease. By Victor C. Vaughan, Ph. D., M. D., Professor of Hygiene and Physiological Chemistry, and Frederick G. Novy, M. D., Junior Professor of Hygiene and Physiological Chemistry in the University of Michigan. New (3d) edition. In one 12mo volume of 603 pages. Cloth, $3.00. Philadelphia: Lea Brothers & Co. 1896.

An American Text-Book of Physiology. By Henry P. Bowditch, M. D., John G. Curtis, M. D., Henry H. Donaldson, Ph. D., W. H. Howell, Ph. D., M. D., Frederic S. Lee, Ph. D., Warren P. Lombard, M. D., Graham Lusk, Ph. D., W. T. Porter, M. D., Edward T. Reichert, M. D., and Henry Sewall, Ph. D., M. D. Edited by William H. Howell, Ph. D., M. D., Professor of Physiology in the Johns Hopkins University, Baltimore, Md. Large octavo, pp. 1052. Illustrated. Price, cloth, $6.00; sheepskin and half Morocco, $7.00. For sale by subscription only. Philadelphia: W. B. Saunders, 925 Walnut street. 1896.

A Practical Treatise on Materia Medica and Therapeutics. By Roberts Bartholow, M. A., M. D., LL. D., Professor Emeritus of Materia Medica, General Therapeutics and Hygiene in the Jefferson Medical College of Philadelphia, etc., etc. Octavo, pp. 866. Ninth edition, revised and enlarged. New York: D. Appleton & Co. 1896.

Report of the Commissioner of Education for the Year 1893-94. Volume I. Containing part 1. Washington: Government Printing Office. 1896.

